# Radical prostatectomy versus external beam radiotherapy with androgen deprivation therapy for high-risk prostate cancer: a systematic review

**DOI:** 10.1186/s12885-023-10842-1

**Published:** 2023-05-04

**Authors:** Berdine L. Heesterman, Katja K. H. Aben, Igle Jan de Jong, Floris J. Pos, Olga L. van der Hel

**Affiliations:** 1 Netherlands Comprehensive Cancer Organisation, Godebaldkwartier 419, 3511 DT Utrecht, The Netherlands; 2 Health Evidence, Radboud University Medical Center, Nijmegen, the Netherlands; 3 Department of Urology, University Medical Center Groningen, Groningen, the Netherlands; 4 Department of Radiation Oncology, The Netherlands Cancer Institute, Amsterdam, the Netherlands

**Keywords:** Prostate cancer, Prostatectomy, Radiotherapy, Systematic Review

## Abstract

**Background:**

To summarize recent evidence in terms of health-related quality of life (HRQoL), functional and oncological outcomes following radical prostatectomy (RP) compared to external beam radiotherapy (EBRT) and androgen deprivation therapy (ADT) for high-risk prostate cancer (PCa).

**Methods:**

We searched Medline, Embase, Cochrane Database of Systematic Reviews, Cochrane Controlled Trial Register and the International Standard Randomized Controlled Trial Number registry on 29 march 2021. Comparative studies, published since 2016, that reported on treatment with RP versus dose-escalated EBRT and ADT for high-risk non-metastatic PCa were included. The Newcastle–Ottawa Scale was used to appraise quality and risk of bias. A qualitative synthesis was performed.

**Results:**

Nineteen studies, all non-randomized, met the inclusion criteria. Risk of bias assessment indicated low (*n* = 14) to moderate/high (*n* = 5) risk of bias. Only three studies reported functional outcomes and/or HRQoL using different measurement instruments and methods. A clinically meaningful difference in HRQoL was not observed. All studies reported oncological outcomes and survival was generally good (5-year survival rates > 90%). In the majority of studies, a statistically significant difference between both treatment groups was not observed, or only differences in biochemical recurrence-free survival were reported.

**Conclusions:**

Evidence clearly demonstrating superiority in terms of oncological outcomes of either RP or EBRT combined with ADT is lacking. Studies reporting functional outcomes and HRQoL are very scarce and the magnitude of the effect of RP versus dose-escalated EBRT with ADT on HRQoL and functional outcomes remains largely unknown.

**Supplementary Information:**

The online version contains supplementary material available at 10.1186/s12885-023-10842-1.

## Background

Radical prostatectomy (RP) and external beam radiotherapy (EBRT) combined with Androgen deprivation therapy (ADT) are widely used treatment modalities for high-risk localized prostate cancer (PCa). To date there is no consensus on which of both is the optimal treatment for men with high-risk PCa, as high-level evidence is lacking [1]. The only high-quality, well-known randomized controlled trial (RCT) comparing RP with EBRT is the ‘Prostate Testing for Cancer and Treatment’ (ProtecT) trial. The purpose of this trial, in which patients were enrolled between 1999 and 2009, was to compare oncological outcomes and side effects of RP, EBRT and active monitoring for, mainly low-risk localized, PCa detected by PSA screening. Only 2% of men included in the ProtecT trial had high-risk PCa [2, 3]. PCa-specific survival (PCSS) was excellent in all treatment groups (approximately 99% at 10 years) and there was no significant difference in PCa-related deaths per 1000 person-years. With respect to functional outcomes, the greatest negative impact was seen after RP and concerned in particular a decline in sexual function and urinary incontinence. Decreased bowel function and irritative urinary symptoms were more often reported following EBRT, but were usually temporary. A difference in general health-related quality of life (HRQoL) was not observed. The results of ProtecT cannot be generalized to high-risk patients, as treatment for high-risk PCa differs from treatment for low- to intermediate-risk PCa. In the latter group, nerve sparing surgery is often possible while this is generally not the case in high-risk PCa. In addition, in men with low- or intermediate-risk PCa treated with EBRT, no ADT or only short-term ADT is advised, while long-term ADT is recommended in case of high-risk PCa [4].

Two small RCTs compared RP with a radiation-based approach in men with localized-locally advanced PCa [5, 6]. Patients were recruited from 1989–1993 and from 1996–2001. No statistically significant differences in PCSS between both treatment groups were observed, however with fewer than 100 patients enrolled in each study, both studies were underpowered for oncological outcomes. In addition, treatment techniques have evolved, therefore results are not generalizable to contemporary practice. Currently, the ‘Scandinavian Surgery Versus Radiotherapy for Locally Advanced Prostate Cancer’ (SPCG-15) trial is the only randomized study comparing RP and EBRT in men with locally advanced PCa. The study is still recruiting and given disappointing accrual it will be some time before endpoints (including PCSS and HRQoL) will be reported [7, 8].

Thus, randomized studies comparing RP with a radiation-based approach are scarce and the existing trials either enrolled a different patient population or were underpowered and are outdated. Next to these randomized trials, multiple observational studies have been published comparing RP with a radiation-based approach in the treatment of high-risk PCa. We conducted a systematic review to summarize the results of recent evidence in terms of HRQoL, functional and oncological outcomes following RP compared to a radiation-based approach in high-risk PCa. In view of advances in surgical and radiation-based treatment of high-risk PCa, we focused on studies published from 2016 onwards.

## Methods

For reporting the results of our review, we followed the Preferred Reporting Items for Systematic Reviews and Meta-analyses (PRIMSA) guidelines (supplementary information p. 1–2) [9] Medline, Embase, Cochrane Database of Systematic Reviews, Cochrane Controlled Trial Register and the International Standard Randomized Controlled Trial Number (ISRCTN) registry were systematically searched on 29 March 2021 for studies published from 2016 onwards. The search strategy is provided in the supplementary information (p. 3). Search results were combined and duplicate publications were removed. Comparative studies (RCTs, cohort and case–control studies) reporting on treatment with RP compared to dose-escalated EBRT and ADT for high-risk nonmetastatic PCa were included if at least 100 patients participated in the study. Patient series without comparison groups, editorials, reviews, commentaries, conference abstracts without publications and articles in languages other than Dutch or English were excluded.

The population of interest consisted of patients of any age, diagnosed with de novo high-risk nonmetastatic PCa. High-risk PCa was defined as ≥ cT2c, cN0/1, cM0, ISUP grade 4–5 and/or PSA > 20 ng/ml. This allowed both studies using the European Association of Urology (EAU) risk classification (high-risk: ≥ cT2c, ISUP grade ≥ 4 or PSA > 20 ng/ml) and studies using the National Comprehensive Cancer Network (NCCN) risk classification (high- or very high-risk: ≥ cT2c, ISUP grade ≥ 4 or PSA > 20 ng/ml) to be included. RP could be performed via an open, laparoscopic or robot-assisted surgical approach, as no approach is currently recommended over another [10]. Furthermore, RP could potentially be part of multimodality therapy with adjuvant RT and/or (neo)adjuvant ADT. Dose-escalated EBRT was defined as a biologically effective dose (BED) converted to 2 Gy fractions (EQD2) of at least 74 Gy. In addition, a brachytherapy boost, could be given [10]. There were no requirements with regard to the radiotherapy technique used (e.g. three-dimensional conformal radiotherapy and intensity modulated radiotherapy). In both treatment groups, pelvic lymph node dissection (PLND) could be performed for staging purposes. The primary outcome measures were HRQoL and functional outcomes. Secondary outcome measures included biochemical recurrence-free survival (BCRFS), clinical recurrence-free survival (cRFS), distant metastasis-free survival (DMFS), PCa-specific survival (PCSS) and overall survival (OS).

Title, abstract and full-text screening were performed independently by OLH and BLH. In case of different assessment, consensus was reached by discussion. For all included studies, details on study design, recruitment period, number of included patients, mean or median age, tumor characteristics (e.g. clinical T-stage, Gleason Score and PSA), treatment details (e.g. surgical approach and radiation dose), mean or median follow-up time and primary and secondary outcome measures were extracted by OLH and/or BLH. The Newcastle–Ottawa Scale was used to appraise the quality and risk of bias of included studies [11, 12]. A follow-up period of 3 years was considered sufficient for the primary outcome measures (HRQoL and functional outcomes), but in case only secondary outcome measures were reported, 5 years was considered the minimum acceptable follow-up length. Appraisal was done independently by OLH and BLH and once again disagreement was resolved by discussion. Studies with a total score of ≥ 7 were considered to have low risk of bias while studies with a score of ≤ 6 were considered to be at moderate to high risk of bias.

## Results

### Study selection process

**Fig. 1:**
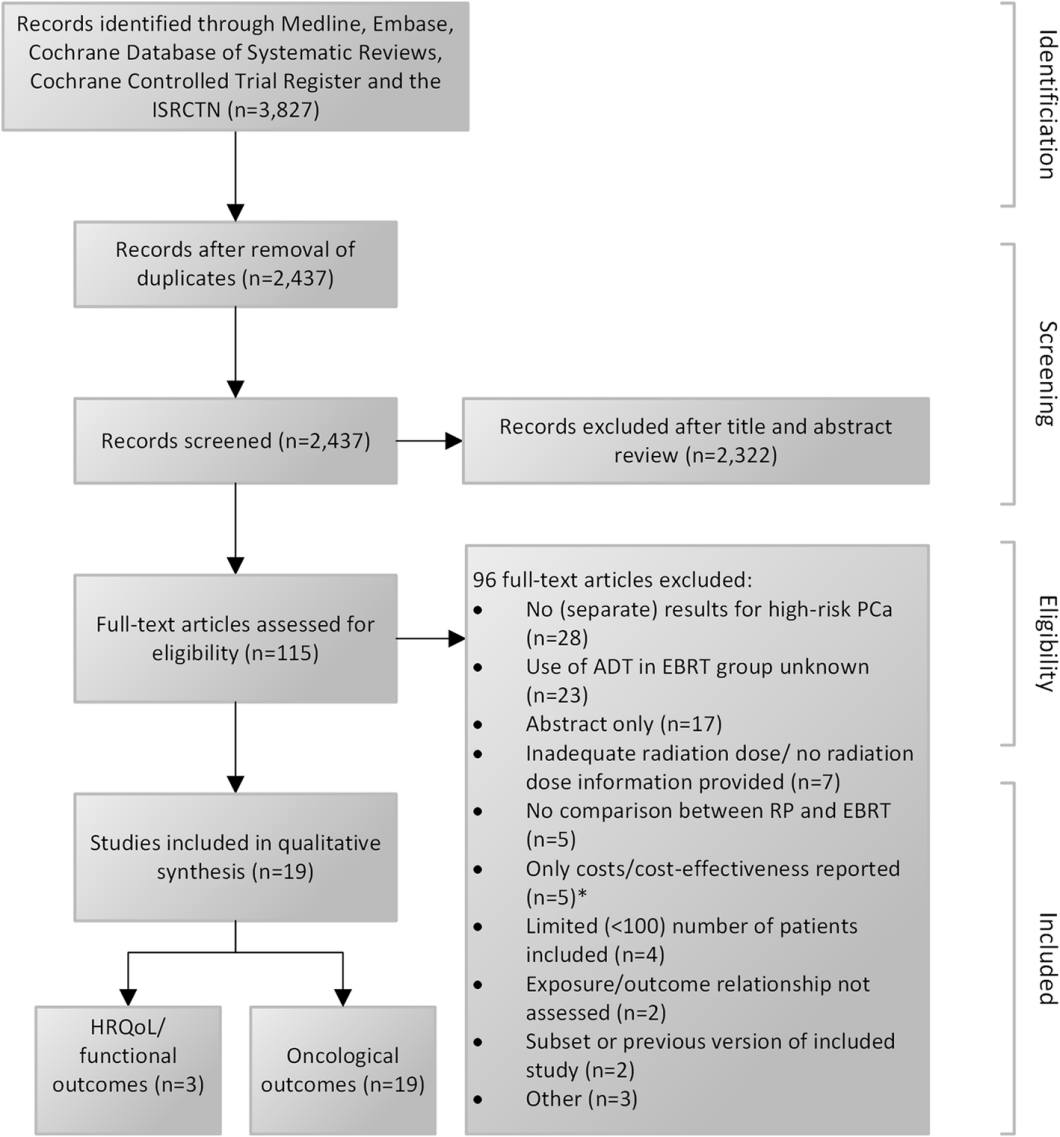
Preferred reporting items for systematic reviews and meta-analyses (PRIMSA) flow diagram. * Studies comparing cost- and/or cost-effectiveness of RP with radiation-based treatment for high-risk PCa, were initially selected as well, but eventually excluded to emphasize patient-relevant outcomes

The study selection process is illustrated in Fig. 1. In total, 3,827 records were identified, of which 2,437 remained after removal of duplicate records. Following title and abstract review, 2,322 records were excluded and 115 full-text articles were assessed for eligibility. Ninety-six full-text articles were excluded, with reasons such as: no (separate) results for high-risk PCa reported, use of ADT in EBRT group unknown, inadequate radiation dose/no radiation dose information provided and abstract only. Finally, 19 studies were included in the qualitative synthesis.

### Narrative description of included studies

All included studies (Table 1) were non-randomized studies, comprising of one prospective population-based cohort study [13], four retrospective population-based cohort studies [14–17], 10 single-institution retrospective cohort studies [18–27], two multicenter retrospective cohort studies [28, 29] and two studies in which data for the two treatment groups came from different (institutional) databases [30, 31]. Both cohort studies for which data were collected retrospectively from electronic medical records and studies that analyzed data from existing (institutional) databases were considered retrospective. The number of included patients varied from approximately 100 to 40,000 and the median follow-up time ranged from approximately 3 to 10 years. Most studies (*n* = 11) used the NCCN definition of high-risk PCa [15–17, 21–27, 29], two studies used the EAU definition [14, 20] and in the remaining studies other definitions were used (e.g. Gleason score ≥ 8) [13, 18, 19, 28, 30, 31]. The mean/median age was approximately 65 years in most studies. However, patients treated with RP were generally younger than patients treated with a radiation-based approach. Information on the surgical approach used was reported in 13 studies [13, 17, 19–27, 29, 31]. In most cases, RP was performed via an open or robot assisted procedure, while a conventional laprascopic approach was less commonly used. The percentage of surgically treated patients who received (neo)adjuvant ADT ranged from 0–36% and the percentage of patients who received adjuvant radiotherapy ranged from 0–44%. Except in one study where a substantially higher percentage of surgically treated patients received (neo)adjuvant therapy (ADT: 60% and radiotherapy: 90%). With regard to the applied radiotherapy technique, information was reported in 11 studies [13, 18–22, 24, 26, 27, 29, 31] and intensity modulated radiotherapy was most often used. The median biologically effective dose (BED) converted to 2 Gy fractions (EQD2) was provided or could be calculated (assuming an α/β of 1.5 Gy and assuming a dose per fraction of 2 Gy in one study where this dose was not reported) in seven studies and ranged from 74-80 Gy. In three studies all patients received an EQD2 ≥ 74 Gy, in seven studies this percentage could not be determined precisely but ranged from 24 up to 100% and in the remaining two studies sensitivity analysis were conducted in a subset of patients who received a radiation dose of ≥ 79 Gy. The percentage of patients treated with ADT in addition to EBRT ranged from 69–100% and exceeded 90% in all but four studies. Three studies reported functional outcomes and/or HRQoL [13, 23, 25] and all studies reported oncological outcomes [13–31].

**Table 1:** Characteristics of included studies

Author (year)	Country, recruitment period	Design	Treatment	N (high-risk)	Age	Gleason score	PSA (ng/ml)	cT	cN	Treatment information	Follow-up time	Outcome measures
Aas (2017) [14]	Norway (2004–2005)	Retrospective cohort (Population database/ cancer registry)	RP	high-risk localized (EAU): *n =* 104 high-risk locally advanced (cT3): *n *= 32	not reported	not reported	not reported	not reported	not reported	RP within 12mos of diagnosis; surgical approach: not reported	median (range): 10 (0–11) yrs	PCSM, OM
			RT ± ADT	high-risk localized (EAU): *n =* 294 high-risk locally advanced (cT3): *n* = 493	not reported	not reported	not reported	not reported	not reported	RT within 18mos of diagnosis with 6mos neoadjuvant ADT; RT technique: not reported; target dose ≥ 70 Gy (70 Gy: 38%, 72 or 74 Gy: 38%, 76 or 78 Gy: 24%; dose per fraction: 2 Gy); (neo)adjuvant ADT lasting for 3 years: 95%	median (range): 10 ( 0–11) yrs	
Andic (2019) [18]	Turkey (Aug 2007-March 2018)	Single-institution retrospective cohort study	RP ± RT ± ADT	high-risk (AUA): *n* = 48	mean (SD): 64.5 (7.6)	> GS8: 30 (62.5%)	≥ 20: 47.9%	cT2: 35 (72.9%) cT3: 12 (25%) cT4: 1 (2.1%)	not reported	RP + PLND; surgical approach: not reported; adjuvant RT:12 (25%) + ADT in 9/12	mean: 41.3 ± 21.5mos	BCRFS, DMFS, PCSS, OS
			EBRT ± ADT	high-risk (AUA): *n* = 72	mean (SD): 67.7 (6.6)	> GS8: 40 (55.6%)	≥ 20: 69.4%	cT2: 46 (63.9%) cT3: 22 (30.6%) cT4: 4 (5.6%)	not reported	3D-CRT: 65 (90.3%); IMRT: 7 (9.7%); median dose (range): 74 Gy (70–76), dose per fraction:1.8-2 Gy; ADT: 95.8% (≥ 2yrs: 65.3%, ≥ 1-2yrs: 25%, < 1 yr: 5.6%)	mean: 60.2 ± 30.3mos	
Baker (2016) [19]	USA (2001–2014)	Single-institution retrospective cohort study	RP ± RT ± ADT	high-risk (GS ≥ 8): *n* = 50	mean: 60.9	≤ GS7: 18 ≥ GS8: 31	mean initial PSA: 11.5 (2.9–50.0)	≤ cT2:: 47 (96%) cT3: 2 (4%)	cNx/cN0: *n* = 49 (98%) cN1: 2 (4%)	ORP (32%)/ RARP (50%)/ unknown surgical approach (18%) ± PLND; (neo)adjuvant: ADT: 18 (36.0%); adjuvant EBRT: 22 (44.0%)	mean: 60mos	BCRFS, DM
			EBRT ± ADT	high-risk (GS ≥ 8) *n* = 71	mean: 69.6	≤ GS7: 0 ≥ GS8: 71	mean initial PSA: 9.58 (1.1–19.0)	≤ cT2: 63 (88.7%) cT3: 8(11.3%)	cNX/cN0: 67 (94.4%) cN1: 4 (5.6%)	3D-CRT or IMRT (percentage missing); total dose 75 to 77 Gy in 40–42 fractions: *n* = 44, total dose 70 to 70.2 Gy in 28 fractions: *n* = 31; ADT: 95.8%	mean: 73.7mos	
Berg (2019) [15]	USA, NCDB, (2004–2009)	Retrospective cohort (Population database/ cancer registry) re-analysis of Ennis et al. with more restrictive inclusion criteria: younger and healthier men who were diagnosed in the earlier study period to ensure sufficient follow-up	RP ± RT ± ADT	high-risk (NCCN) *n* = 12,283	median: 58.15 (exclusion age ≥ 66 yr)	≤ GS6: 1757 GS7: 3449 GS8: 3777 GS9: 3184 GS10:116	< 10: 6032 10–20: 1550 > 20: 4701	cT1: 6391 cT2: 3697 cT3: 2111 cT4: 84	all N0	Surgical approach: not reported; (neo)adjuvant ADT: 15%; adjuvant RT: 15%	median: 91.0mos	OS
			EBRT + BT ± ADT	high-risk (NCCN): *n* = 1702	median: 58.12 (exclusion age ≥ 66 yr)	≤ GS6:202 GS7:394 GS8:717 GS9: 359 GS10:30	< 10: 795 10–20: 273 > 20: 634	cT1: 745 cT2: 611 cT3: 340 cT4: 6	all N0	RT technique: not reported, dose not reported (sensitivity analysis reported by Ennis et al. with 2 groups: < 7920 cGy versus ≥ 7920 cGy); ADT: 1176 (69%)	median: 101mos	
Cano-Velasco (2019) [20]	Spain (1996–2008)	Single-institution retrospective cohort study	RP	high-risk (EAU): *n* = 145	median: 65	≤ GS6:19 (13.1%) GS7: 14 (9.7%) ≥ GS8: 112 (77.2%)	> 20: 30 (20.7%)	cT1: 48 (33.1%) cT2a-b: 59 (40.7%) cT2c: 38 (26.2%) cT3a: 0 (0)	not reported	ORP; RP (monotherapy) ± PLND	median: 152mos	PCSS, OS
			EBRT + ADT	high-risk (EAU): *n* = 141	median: 71	≤ GS6: 26 (18.4%) GS7: 49 (34.8%) ≥ GS8: 66 (46.8%)	> 20: 66 (46.8%)	cT1: 24 (17%) cT2a-b: 32 (22.7%) cT2c: 64 (45.4%) CT3a: 21 (14.9%)	not reported	3D-CRT; median total dose (IQR): 74 Gy (74–75); dose per fraction: not reported; ADT: 100%	median: 97mos	
Ciezki (2017) [25]	USA (1996–2012)	Single-institution retrospective cohort study	RP ± RT ± ADT	high-risk (NCCN): *n* = 1308	median: 62	GS6:70 GS7:662 GS8:397 GS9:178 GS10:4	≥ 20: 196 (15%)	≤ cT2: 1268 cT3: 43	not reported	ORP (56%); LRP (8%); RARP (36%); (neo)adjuvant ADT:19%; adjuvant or salvage RT: 18.6%	median: 55.6mos	BCRFS, cRFS, PCSM, GI and GU toxicity (EHR data)
			EBRT ± ADT	high-risk (NCCN): *n* = 734	median: 68.5	GS6:76 GS7:354 GS8:178 GS9:117 GS10:9	≥ 20: 271 (36%)	≤ cT2: 633 cT3: 101	not reported	RT technique: not reported; dose: ≥ 78 Gy at 2 Gy/fraction (52%) or 70 Gy at 2.5 Gy/fraction (48%); (neo)adjuvant ADT: 93% (> 6mos: 26%; 1-6mos:66%)	median: 94.6mos	
Emam (2020) [26]	USA (March 2006-July 2017)	Single-institution retrospective cohort study	RP ± RT ± ADT	high- or very high-risk localized PCa (NCCN): *n* = 291	mean: 61	GS6: 8 GS7: 60 GS8:142 GS9-10:81	highest pretreatment PSA: 7.89	cT1: 129 cT2: 129 cT3: 33	not reported	RARP + PLND (97%); neoadjuvant ADT: 22 (7.6%); adjuvant/salvage therapy: 170 (58%); adjuvant/salvage EBRT:135 (46%); ADT: 91 (31%)	median (range): 5.1 (2.3–12.8) yrs, cases with less than 2yrs follow-up were excluded	BCRFS, DMFS, PCSS, OS
	USA (April 2007-Oct 2017)		EBRT ± ADT	high- or very high-risk localized PCa (NCCN): *n* = 44	mean: 71	GS6:0 GS7: 4 GS8: 26 GS9-10:14	highest pretreatment PSA: 10.58	cT1: 19 cT2: 23 cT3: 1	not reported	VMAT; median total dose 81 Gy in 45 fractions; (neo)adjuvant ADT: 42 (95%), median (IQR) duration: 24 (18)mos	median (range): 3.3 (2–12.4) yrs, cases with less than 2yrs follow-up were excluded	
Ennis (2018) [16]	USA (NCDB: 2004–2013)	Retrospective cohort (Population database/ cancer registry)	RP	high-risk (NCCN): *n* = 24,688	mean (SD): 62.61 (7.02)	≤ GS6: 2,652 (10.74%) GS7: 4,705 (19.06%) GS8: 11,081 (44.88%) GS9: 5,910 (23.94%) GS10: 340 (1.38%)	mean (SD): 19.02 (21.13)	≤ cT2: 21,968 (88.97%) ≥ cT3: 2,723 (11.03%)	all N0	Surgical approach: not reported	only for the total group: median 36.34mos	OS
			EBRT + ADT	high-risk (NCCN): *n* = 15,435	mean (SD): 69.66 (8.19)	≤ GS6: 553 (3.58%) GS7: 2,837 (18.38%) GS8: 6,545 (42.40%) GS9: 4,968 (32.19%) GS10: 532 (3.45%)	mean (SD): 22.58 (23.81)	≤ cT2: 12,906 (83.62%) ≥ cT3: 2,723 (11.03%)	all N0	RT technique: not reported; sensitivity analysis with 2 groups: < 7920 cGy versus ≥ 7920 cGy; ADT: 100%		
			EBRT + BT ± ADT	high-risk (NCCN): *n* = 2,642	mean (SD): 67.15 (7.72)	≤ GS6:171 (6.47%) GS7: 546 (20.67%) GS8: 1,190 (45.04%) GS9: 683 (25.85%) GS10: 52 (1.97%)	mean (SD): 18.96 (20.75)	≤ cT2: 2,233 (84.52%) ≥ cT3: 409 (15.48%)		RT technique: not reported; sensitivity analysis with 2 groups: < 7920 cGy versus ≥ 7920 cGy		
Gunnarsson (2019) [30]	Sweden (1995–2010)	Retrospective cohort study: RP from single institution; RT from National Prostate Cancer Register (NPCR)	RP ± RT ± ADT	high-risk (modification of D'Amico criteria: cT3 and/or PSA 20–50 ng/ml and /or GS 8–10): *n* = 153	mean: 64.2	≤ GS6: 27 GS7 53 GS8-10: 73	mean: 19.3	≤ cT2: 101 ≥ cT3: 52	not reported	Surgical approach: not reported; PLND: 135 (88%); neoadjuvant ADT: 131 (86%); adjuvant ADT: 11 (7%); adjuvant RT: 99 (64%)	2yrs: 100%; 5yrs: 95%; 10yrs: 87%; 15yrs: 84%	PCSS, OS
			EBRT ± BT ± ADT	high-risk (modification of D'Amico criteria): *n* = 702	mean: 64.3	≤ GS6: 152 GS7: 305 GS8-10: 245	mean: 20.6	≤ cT2: 329 ≥ cT3: 371	not reported	RT technique: not reported; EBRT up to 78 Gy alone: 495 (71%); HDR-BT 20 Gy + EBRT 50 Gy: 207 (29%); RT with neoadjuvant ADT was preferred treatment; ADT usually prolonged up to 2yrs after RT	2yrs: 99%; 5yrs: 94%; 10yrs: 84%; 15yrs: 70%	
Hayashi (2020) [21]	Japan (2004–2015)	Single-institution retrospective cohort study	RP ± ADT	total: *n* = 462; high-risk (NCCN): *n* = 163	66 ± 6.1 (*n* = 462)	GS 8–10: 106	8.9 ± 10.5 (*n* = 462)	cT2c: 63 cT3-4: 32	not reported	2004–2011: ORP; 2011–2012: ORP or LRP; 2014–2015: RARP ± PLND; ADT: 23 (5%)	median (range): 77 (13.3–155) mos (*n* = 462)	BCRFS, OS
			EBRT ± ADT	total: *n* = 319; high-risk (NCCN): *n* = 174	73 ± 5.5 (*n* = 319)	GS 8–10: 93	11.2 ± 15.2 (*n* = 319)	cT2c: 35 cT3-4: 74	not reported	IMRT; dose 2004–2006: 74.7 Gy/37 fractions—82.3 Gy/42 fractions; dose 2006–2015: 76 Gy/38 fractions; ADT: 98.1%, median (range) duration: 35 (2–96) mos	median (range): 54 (1.9–143) mos (*n* = 319)	
Hoffman (2020) [13]	USA (2011–2012)	Prospective population based cohort study	RP	unfavorable risk (cT2cN0M0 PSA 20–50 ng/ml; Grade group 3, 4 or 5): *n* = 402	median: 64 (IQR 59–68)	≤ GS7: 252 (63%) GS8-10: 149 (37%)	median (IQR): 6 (5–9)	cT1: 212	cN0	RARP: 257 (66%)	median (IQR): for vital status: 73(63–79) mos (favorable and unfavorable)	EPIC score, SF-36 score, PCSS, OS
			EBRT + ADT	unfavorable risk (cT2cN0M0 PSA 20–50 ng/ml; Grade group 3, 4 or 5): *n* = 217	median: 71 (IQR 66–74)	≤ GS7: 118 (54%) GS8-10: 98 (45%)	median (IQR): 7 (5–13)	cT1: 124	cN0	IMRT: 188 (87%), median (IQR) total dose: 78 Gy (76-79 Gy); median (IQR) dose per fraction: 1.8 (1.8–2.0); ADT: 100%	median (IQR): for vital status: 73(63–79) mos (favorable and unfavorable)	
Kishan (2018) [28]	USA, Norway (2000–2013)	Multicenter retrospective cohort study	RP ± RT ± ADT	high-risk (GS = 9–10): *n* = 639	median: 61.0	GS9: 613 GS10: 26	mean (range): 11.26 (0.4–378.6)	≤ cT2: 557 (87%) cT3a: 36 (6%) cT3b: 21 (3%) cT4: 24 (3%)	not reported	Surgical approach: not reported; neoadjuvant systemic therapy: 19%; adjuvant RT: 8.7%; adjuvant systemic therapy: 11.3%	median: 4.2yrs	DM, PCSM, OS
			EBRT ± ADT	high-risk (GS = 9–10):*n* = 734	median: 67.7	GS9: 686 GS10: 48	mean (range): 21.5 (0.4–525.5)	≤ cT2 412 (56%) cT3a: 103 (14%) cT3b: 75 (10%) cT4: 44 (6%)	not reported	RT technique: not reported; dose: median (range) EQD2 (assuming an α/β of 1.5 Gy): 74.3 Gy (65–81.4); ADT: 89.5% (median duration 21.9mos)	median: 5.1yrs	
			EBRT + BT ± ADT	high-risk (GS = 9–10): *n* = 436	median: 67.5	GS9: 398 GS0: 38	mean (range): 14.8 (0.1–273.5)	≤ cT2: 343 (78%) cT3a: 63 (14%) cT3b: 7 (2%) cT4: 3 (1%)	not reported	RT technique: not reported; dose: median EQD2 (range) 91.5 Gy (75.8–131.4); ADT: 92.4% (median duration: 12mos)	median: 6.3yrs	
Koo (2018) [29]	Korea (2000–2016)	Multicenter retrospective cohort study	RP	total: *n* = 339; high-risk (NCCN): *n* = 209	median (IQR): 70.0 (66–73) (*n* = 339)	≤ GS7: 78 (23%); GS 7: 133 (39.2%); GS:8–9: 128 (37.8%)	median (IQR): 10.4 (6.7–20.7) (*n* = 339)	≤ cT2: 219 (64.6%) cT3: 99 (29.2%) cT4: 21 (6.2%)	cN0: 322 (95%) cN1: 17 (5%)	Retropubic/RARP ± PLND	median (IQR): 69.0 (42.7–94.0) mos	BCRFS, DMFS, PCSS, OS
			EBRT ± ADT	total: *n* = 339; high-risk (NCCN): *n* = 209	median (IQR): 70.1 (66–74) (*n* = 339)	≤ GS7: 78 (23%); GS 7: 133 (39.2%); GS 8–9: 128 (37.8%)	median (IQR):10.7 (7.0–21.5) (*n* = 339)	≤ cT2: 219 (64.6%) cT3: 99 (29.2%) cT4: 21 (6.2%)	cN0: 322 (95%) cN1: 17 (5%)	Overall: 3D-CRT: 216 (63.7%); IMRT: 123 (36.3%); median (IQR) total dose: 70 Gy (70-74 Gy), in 33.5 fractions (IQR: 28–37), dose > 76 Gy: 295 (87%); ADT: 186 (88.9%)	median (IQR): 60.5 (39.0–98.0) mos	
Markovina (2017) [22]	USA (2001–2011)	Single-institution retrospective cohort study	RP ± RT ± ADT	high-risk (NCCN); *n* = 62	mean (SD): 62.9 (7.1)	GS 6–7: 17 (27.4%) GS 8: 30 (48.4%) GS 9–10: 15 (24.2%)	< 10: 30 (48.4%) 10–20: (8.1%) > 20: 27 (43.5%)	≤ cT2: 59 (95.2%) cT3: 3 (4.8%)	not reported	ORP, LRP or RARP + PLND; adjuvant RT and/or ADT: 5 (8%)	median (± SD): 41 ± 26.5mos	DMFS, OS
			EBRT ± ADT	high-risk (NCCN): *n* = 62	mean (SD): 64.2 (9.1)	GS 6–7: 17 (27.4%) GS 8: 30 (48.4%) GS 9–10: 15 (24.2%)	< 10: 30 (48.4%) 10–20: (8.1%) > 20: 27 (43.5%)	≤ cT2: 59 (95.2%) cT3: 3 (4.8%)	not reported	IMRT: 60 (97%); median (range) total dose: 75.6 Gy (73.8–77.4); ADT: 80.6%	median (± SD): 51.4 ± 29.8mos	
Reichard (2019) [27]	USA (2004–2013)	Single-institution retrospective cohort study	RP ± RT ± ADT	high-risk (NCCN): *n* = 231	median (range): 61 (41–80)	≤ GS7: 33 (14%) GS 8: 115 (50%) GS9-10: 83 (36%)	median (range): 6.8 (1–36)	≤ cT2: 177 (77%) cT3-4: 54 (23%)	not reported	ORP: 130 (56%); RARP: 101 (44%); PLND: 100%; neoadjuvant ADT: 73 (32%), median (IQR) duration: 3 (1–14) mos; adjuvant RT: 9 (4%)	median (range): 79 (1–155) mos	BCR, LR, DMF,OS
			RT + ADT	high-risk (NCCN): *n* = 73	median (range): 66 (54–78)	≤ GS7: 7: 15 (21%) GS 8: 31 (43%) GS9-10: 27 (37%)	median (range): 6.8 (1–29)	≤ cT2: 51 (70%) cT3-4: 22 (30%)	not reported	IMRT (85%), Proton (12%), VMAT (3%); dose > 75 Gy in 99%, ≥ 74 Gy in 100%; ADT: 100%, median (IQR) duration: 22mos (14–23)	median (range): 87 (20–149) mos	
Robinson (2018) [17]	Sweden (1998–2012)	Retrospective cohort (Population database/ cancer registry)	RP	total: *n* = 26,449; high-risk (NCCN): *n* = 3321	mean (SD): 63.1 (5.8) (*n* = 26,449)	ISUP ≤ 3: 23,283 (88%) ISUP 4–5: 1770 (7%)	median (IQR): 6.9 (4.9–10) (*n* = 26,449)	≤ cT2: 25,483 (96%) T3: 745 (3%)	cN0: 5545 (21%) cNx: 20778 (79%)	1998–2002: 3462 (81.0%) ORP, 426 (14.4%) LRP, 1684 (4.6%) RARP; 2003–2008: 6810 (70.3%) ORP, 807 (7.3%) LRP, 3328 (22.3%) RARP; 2009–2012: 6181 (43.7%) ORP, 734 (4.6%) LRP, 3017 (51.8%) RARP	mean (± SD): 7.3 (± 3.7) yrs	PCSM
			EBRT ± ADT	total: *n* = 15,504; high-risk (NCCN): *n* = 6041	mean (SD): 67 (5.8) (*n* = 15,504)	ISUP ≤ 3: 11,632 (75%) ISUP 4–5: 2487 (16%)	median (IQR): 10 (6.4–18) (*n* = 15,504)	≤ cT2: 11,814 (76%) T3: 3101 (21%)	cN0: 4498 (30%) cNx: 10470 (70%)	RT technique: not reported; 14,512 (94%) EBRT ± HDR-BT/ photon/ proton boost, 992 (6%) BT; Median EQD2 (α/β = 3 Gy) EBRT/HDR-BT ± EBRT: 1998–2002: 71.9 Gy/101.7 Gy, 2003–2008: 77.0 Gy/101.3 Gy and 2009–2012: 78.8 Gy/101.4 Gy; ADT: 90% (≥ 2006)	mean (± SD): 6.9 (± 3.7) yrs	
Tilki (2019) [31]	USA and Germany (1992–2013)	Retrospective cohort study: RP cohort from single institution in de USA; EBRT cohort from single institution in Germany	RP ± EBRT ± ADT	high-risk (GS9-10): *n* = 559	median (IQR): RP: 66.40 (60.81–70.46) RP + EBRT: 66.64 (61.83–69.81) RP + ADT: 66.38 (61.48–69.98) maxRP: 66.04 (61.69–69.67)	GS9: 556 (99%) GS10: (1%)	median (IQR): RP: 12.02 (8.18–22.99); RP + EBRT: 12.50 (6.98–22.65); RP + ADT: 21.00 (11.00–39.49); maxRP: 13.11 (8.40–32.68)	≤ cT2: 538 (96%) cT3-4: 21 (4%)	All cN0	RP: 372 (66.5%); RP + EBRT: 88 (15.7%); RP + ADT:49 (8.8%); RP + EBRT + ADT (maxRP): 50 (8.9%); ORP: 92.8%, RARP: 7.2%; PLND: 100%	median (IQR): 4.78 (4.01–6.05) yrs	PCSM, ACM
			EBRT + BT + ADT (maxRT)	high-risk (GS9-10): *n* = 88	median (IQR): 70.34 (64.18–74.23)	GS9: 75 (94%) GS10: 5 (6%)	median (IQR):10.55 (6.58–18.38)	≤ cT2: 47 (59%) cT3-4: 33 (41%)	All cN0	IMRT; EBRT dose: 25 fractions of 1.8 Gy; BT (I-25,Pd or Cs-131): 90-108 Gy; ADT: 100%, median (IQR) duration: 6 (4–12) mos	median (IQR): 5.51 (2.19–6.95) yrs	
Tward (2020) [23]	USA (2000–2017)	Single-institution retrospective cohort study	RP ± RT ± ADT	high-risk and very high-risk (NCCN): *n* = 410	63.9	ISUP1-3: 27% ISUP4: 45% ISUP5: 29%	< 10: 42% 10–20: 32% > 20: 26%	T1-T2: 88% T2-T3:12%	not reported	RARP: 218 (53.2%); nerve-sparing surgery 293 (71.5%); PLND: 331 (80.7%); adjuvant/salvage RT: 141 (34%), combined with ADT in 73%, median duration: 6mos	median: 4.2yrs for OS and DMFS	HRQoL (SHIM, AUA-SS, IPSS, SHIM, RFAS after 2003) DMFS, OS
			EBRT + ADT	high-risk and very high-risk (NCCN): *n* = 90	69	ISUP1-3: 31% ISUP4: 42% ISUP5: 27%	< 10: 26% 10–20: 41% > 20: 33%	T1-T2: 76% T2-T3:24%	not reported	RT technique: not reported; dose: median (range) EQD2: 75.2 Gy (71.8–83.8); ADT: 100%, median (IQR) duration: 18 (6–28) mos	median: 7.3yrs and 6.3yrs for OS and DMFS	
			EBRT + BT + ADT	high-risk and very high-risk (NCCN): *n* = 86	69.4	ISUP1-3: 31% ISUP4: 42% ISUP5: 27%	< 10: 36% 10–20: 37% > 20: 27%	T1-T2: 59% T2-T3:41%	not reported	RT technique: not reported; median EQD2: 44.4 Gy + Brachy (LDR: 64%, HDR: 36%); ADT: 100%, median (IQR) duration: 6 (6–6) mos	median: 7.0yrs and 5.6yrs for OS and DMFS	
Yamamoto (2016) [24]	Japan (2007–2013)	Single-institution retrospective cohort study	RP	high-risk (NCCN): *n* = 71	median (range): 70 (56–82)	≤ GS7: 35 (49.3%) GS8-10: 36 (50.7%)	median (range): 11.9 (4.3–63.9)	≤ cT2: 34 (48%) cT3a: 37 (52%)	all cN0	ORP + PLND; patients who received concurrent ADT and or adjuvant RT were excluded	median (range): 59.1 (9.0–106.9) mos	BCRFS
			EBRT + ADT	high-risk (NCCN): *n* = 43	median (range): 73 (58–83)	≤ GS7:: 7 (16.3%) GS8-10: 36 (83.7%)	median (range): 17.6 (4.7–204)	≤ cT2: 24 (56%) cT3a: 19 (44%)	all cN0	2007–2010: 3D-CRT (70 Gy in 35 fractions); 2010–2013: VMAT (78 Gy in 39 fractions); ADT: 100%, median (range) duration: 21.4 (9.2–28.9) mos	median (range): 54.5 (29.2–107) mos	

*Abbreviations*: *ACM* All-cause mortality, *ADT* Androgen Deprivation Therapy, *AUA-SS* American Urological Association Symptom Score, *BCRFS* Biochemical Recurrence-Free Survival, *BT* Brachytherapy, *cRFS* Clinical Relapse-Free Survival, *3D-CRT* three-dimensional conformal radiotherapy, *DMFS* Distant Metastases-Free Survival, *EBRT* External Beam Radiotherapy, *EHR* Electronic Health Records, *EPIC* Expanded Prostate Cancer Index Composite, *GI* Gastrointestinal, *GU* Genitourinary, *HRQoL* Health related quality of life, *IPSS* International Prognostic Scoring System, *ISUP* International Society of Urological Pathology, *LRP* Laparoscopic Radical Prostatectomy, *LR* Local Recurrence, *OM* Overall Mortality, *OS* Overall Survival, *PCSM* Prostate Cancer-Specific Mortality, *PCSS* Prostate Cancer-Specific Survival, *RARP* Robot-Assisted Radical Prostatectomy, *RFAS* Rectal Function Assessment Scale, *RP* Radical Prostatectomy, *RT* Radiotherapy, *SF-36* Short Form-36, *SHIM* Sexual Health Inventory in Men, *VMAT* Volumetric Arc Therapy

### Risk of Bias

Appraisal using the Newcastle–Ottawa Scale indicated low risk of bias in 14 studies and moderate to high risk of bias in the remaining five studies (Table 2). Regarding patient selection, it should be noted that the selection of the RP and EBRT cohorts differed in three studies, potentially introducing selection bias [26, 30, 31]. In these three studies the RP and EBRT cohorts were selected from different sources (institutional database versus national cancer registry), different hospitals or different exclusion criteria were applied. With respect to comparability between both treatment groups, most studies (*n* = 15) used some method to control for potential confounders and of those, nine studies used a propensity score method. Nevertheless, bias due to residual and/or unmeasured confounding will still be an issue. Most potential quality issues that were encountered, were related to the assessment of outcome(s), the follow-up length or the adequacy of follow-up. Details on how the outcome (e.g. distant metastases-free survival) was assessed was often lacking or not clearly described (*n* = 10). Follow-up length was insufficient (*n* = 7) or little information was provided on follow-up schedules and/or completeness of follow-up (*n* = 12).

**Table 2:**
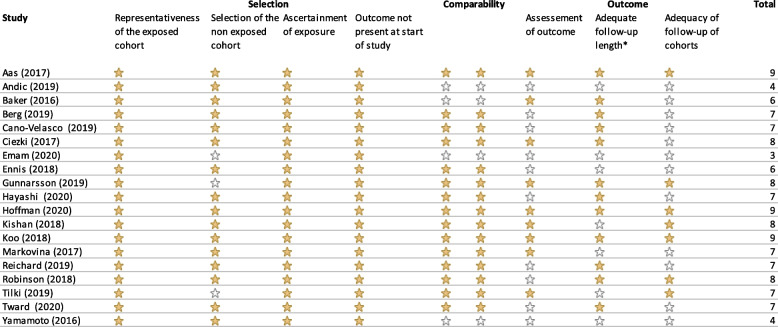
Newcastle-Ottawa scale for risk of bias assessment of included studies

*A follow-up period of 3 years was considered sufficient for the primary outcome measures (HRQoL and functional outcomes), but in case only secondary outcome measures (e.g. BCRFS, cRFS, OS) were reported, 5 years was considered the minimum acceptable follow-up length

### Health-related quality of life and functional outcomes

Three studies reported functional outcomes and/or HRQoL, collected in different ways [13, 23, 25]. In the first study, patient reported outcome measures (PROMS) were collected prospectively [13]. In the second, historic cohort study, PROMS were collected during routine clinical care and available for approximately 50% of the study population [23]. The third study reported 10-year cumulative incidence of ≥ grade 3 genitourinary (GU) and gastrointestinal (GI) toxicity (defined according to the Common Terminology Criteria for Adverse Events version 4.03) and retrieved the information from electronic health records [25]. Despite the use of different measurement instruments and methods, in general it can be concluded that GU toxicity and sexual dysfunction were more often reported after RP (Table 3). In contrast, GI toxicity was more often reported after EBRT, although reported differences were not clinically relevant in all studies. In addition, hormonal function was reduced during treatment with ADT [13, 23, 25]. With regard to general HRQoL, measured with the short from (SF)-36 validated questionnaire, a clinically important difference between RP and EBRT combined with ADT was not observed [13].

**Table 3:** Primary outcome measures in included studies

Author (year)	Treatment	Outcome: HRQoL
Ciezki (2017) [25]	RP ± RT ± ADT	10 yr cumulative incidence of ≥ grade3 GU and GI toxicity: 16.4% and 1.0%
	EBRT ± ADT	10 yr cumulative incidence of ≥ grade3 GU and GI toxicity: 8.1% and 4.6%
Hoffman (2020) [13]	RP	EPIC: EBRT + ADT vs RP at 3yrs (higher scores indicate a better function):
		- Sexual function score: 9.1 (3.5–14.8) – MCID: 10–12
		- Urinary incontinence score: 21.8 (17.1–26.6) – MCID: 6–9
		- Urinary Irritative score: 1.1 (-1.6; 3.7) – MCID: 5–7
		- Bowel function score: -1.6 (-4.3;1.2) – MCID: 4–6
		- Hormone function score: -0.2 (-3.0; -2.6) – MCID: 4–6
		SF-36: EBRT + ADT vs RP at 3yrs (higher scores indicate a better function):
		- Physical Function score: -4.8 (-9.0; -0.7) – MCID: 7
		- Emotional Wellbeing score: -1.7 (-4.4; 1.1) – MCID: 6
		- Energy/Fatigue score: -3.4 (-6.7; -0.2) – MCID: 9
	EBRT + ADT	see RP
Tward (2020) [23]	RP ± RT ± ADT	Difference from baseline at 24–42 months: SHIM (sexual dysfunction, lower is worse): -10 AUA-SS (urinary obstruction and irritation, higher is worse):-2.6 RFAS (bowel problems, higher is worse): 1.6
	EBRT + ADT	Difference from baseline at 24–42 months: SHIM:-10.5, AUA-SS: -0.7; RFAS: 2.0
	EBRT + BT + ADT	Difference from baseline at 24–42 months: SHIM:-7, AUA-SS: -1.4; RFAS: 1.7

*Abbreviations*: *ADT* Androgen Deprivation Therapy, *BT* Brachytherapy, *EPIC* Expanded Prostate Cancer Index Composite, *GI* Gastrointestinal, *GU* Genitourinary, *HRQoL* Health related quality of life, *MCID* Minimal Clinically Important Difference, *RFAS* Rectal Function Assessment Scale, *RP* Radical Prostatectomy, *RT* Radiotherapy, *SF-36* Short Form-36, *SHIM)* Sexual Health Inventory in Men

### Oncological outcomes

All 19 studies reported oncological outcomes. In both treatment groups, PCSS and OS were generally good, with most studies reporting five-year OS and PCSS rates of well over 90% (Table 4). With regard to differences in oncological outcomes between surgery and radiation-based treatment, results varied. Most studies (*n* = 6) concluded that surgical and radiation-based treatment are similar with respect to oncological outcomes [13, 17, 20, 23, 29, 31], or only reported more favorable BCRFS (*n* = 5) after treatment with EBRT and ADT (no difference in DMFS/PCSS/OS) [18, 21, 24, 26, 27]. Four studies reported more favorable results after RP compared to EBRT with ADT [14–16, 25], although in one of these studies this was no longer the case when RP was compared to EBRT and brachytherapy (with or without ADT) [16]. Two studies reported more favorable results after EBRT with ADT versus RP [19, 22]. Kishan et al. concluded that treatment with EBRT, brachytherapy and ADT was preferred over RP and over EBRT with ADT [28]. Finally, Gunnarson et al. observed better survival outcomes after triple treatment with RP, EBRT and ADT compared to EBRT with ADT [30].

**Table 4:** Secondary outcome measures in included studies

Author (year)	Outcome measures	Treatment	Oncological outcome
Aas (2017) [14]	PCSM, OM	RP	10 yr PCSM (95%CI): Localized: 4.9% (1.8–10.2), Advanced: 6.5% (1.1–18.2); 10 yr OM (95%CI): Localized: 17.8% (11.6–26.8), Advanced: 9.7% (3.2–27.1)
		RT ± ADT	10 yr PCSM (95%CI):Localized: 7.6% (4.9–11.1), Advanced: 9.2% (6.8–12.0); 10 yr OM (95%CI): Localized: 20.1% (15.9–25.2), Advanced: 24.5% (20.9–28.6)
Andic (2019) [18]	BCRFS, DMFS, PCSS, OS	RP ± RT ± ADT	5 yr BCRFS (95%CI): 38.5% (20.1–56.9); 5 yr DMFS (95%CI): 90.9% (82.4–99.4); 5 yr PCSS(95%CI): 96.9% (90.8–100.0); 5 yr OS (95%CI): 87.2% (76.6–97.9)
		EBRT ± ADT	5 yr BCRFS (95%CI): 78.1% (66.7–89.5); 5 yr DMFS (95%CI): 89.5% (81.4–97.6); 5 yr PCSS (95%CI): 94.1% (87.2–100.0); 5 yr OS (95%CI): 86.8% (77.2–96.3)
Baker (2016) [19]	BCRFS, DM	RP ± RT ± ADT	5 yr DM rate: 7.8%; Cheng et al.: EBRT vs RP: 5 yr BCRFS: 57.7%, HR = 0.35 (0.11–1.13)
		EBRT ± ADT	5 yr DM rate: 2%, Cheng et al.: 5 yr BCRFS: 92.8%
Berg (2019) [15]	OS	RP ± RT ± ADT	RP vs EBRT + BT: HR (95%CI): 0.82 (0.70–0.96)
		EBRT + BT ± ADT	EBRT + BT vs RP: HR (95%CI): 1.22 (1.05–1.43)
Cano-Velasco (2019) [20]	PCSS, OS	RP	5 yr PCSS: 95.7%; 5 yr OS: 92.4%; RP vs EBRT- HR (95%CI):0.48 (0.48–1.50)
		EBRT + ADT	5 yr PCSS 97%; 5 yr OS: 89.2%
Ciezki (2017) [25]	BCRFS, cRFS, PCSM, GI and GU toxicity (EHR data)	RP ± RT ± ADT	5 yr BCRFS (95%CI): 65% (61–68); 5 yr cRFS (95%CI): 89% (86–91); 5 yr PCSM (95%CI): 2.8% (1.7–3.9); bRFS—RP vs EBRT: HR (95%CI): 1.43 (1.19–1.79); cRFS—RP vs EBRT: HR (95%CI): 0.72 (0.54–0.97); PCSM—RP vs EBRT: HR (95%CI): 0.50 (0.32–0.77)
		EBRT ± ADT	5 yr BCRFS (95%CI): 74% (70–77); 5 yr cRFS (95%CI): 85% (83–88); 5 yr PCSM (95%CI): 5.3% (3.6–7.1)
Emam (2020) [26]	BCRFS, DMFS, PCSS, OS	RP ± RT ± ADT	5 yr BCRFS 36%; 5 yr DMFS 77%; 3 yr PCSS 98%; 3 yr OS 97%
		EBRT ± ADT	5 yr BCRFS 75%; 5 yr DMFS 91%; 3 yr PCSS 98%; 3 yr OS 94%
Ennis (2018) [16]	OS	RP	See EBRT + ADT/ EBRT + BT ± ADT
		EBRT + ADT	EBRT + ADT vs RP: HR (95%CI):1.53 (1.22–1.92) and 1.33 (1.05 -1.68) in the ≥ 7920 cGy subgroup
		EBRT + BT ± ADT	EBRT + BT ± ADT vs RP: HR (95%CI):1.17 (0.88–1.55)
Gunnarsson (2019) [30]	PCSS, OS	RP ± RT ± ADT	5 yr PCSS: 95.3%, 5 yr OS: 90.8%; At the end of the study period PCSM was 10%
		EBRT ± BT ± ADT	5 yr PCSS 94.3%, 5 yr OS: 90.7%; At the end of the study period the PCSM was 15%; HR (95%CI): 2.01(1.17–3.43), *p* = 0.011
Hayashi (2020) [21]	BCRFS, OS	RP ± ADT	BCRFS improved in EBRT compared to RP group (p < 0.001); OS: no statistically significant difference
		EBRT ± ADT	See RP
Hoffman (2020) [13]	EPIC score, SF-36 score, PCSS, OS	RP	5 yr PCSS: 99.5% (98.8, 100); 5 yr OS: 97.7% (96.2, 99.2)
		EBRT + ADT	5 yr PCSS: 99.0% (97.7, 100); 5 yr OS: 91.8% (88.2, 95.6)
Kishan (2018) [28]	DM, PCSM, OS	RP ± RT ± ADT	See EBRT + ADT / EBRT + BT ± ADT
		EBRT ± ADT	DM: EBRT vs RP HR (95%CI): 0.90(0.70–1.14); PCSM: EBRT vs RP HR (95%CI): 0.92 (0.67–1.26); OS: EBRT vs RP, ≤ 7.5 yr: HR(95%CI): 1.07 (0.80–1.44); > 7.5 yr: HR (95%CI): 1.34 (0.85–2.11)
		EBRT + BT ± ADT	DM: EBRT + BT vs RP HR (95%CI): 0.27 (0.17–0.43); PCSM: EBRT + BT vs RP HR (95%CI): 0.38 (0.21–0.68); OS: EBRT + BT vs RP ≤ 7.5 yr: HR (95%CI): 0.66 (0.46–0.96), > 7.5 yr: HR (95%CI): 1.16 (0.70–1.92)
Koo (2018) [29]	BCRFS, DMFS, PCSS, OS	RP	5 yr BCRFS: 3.7%; 5 yr DMFS: 33.3%;5 yr PCSS: 98%; 5 yr OS: 93.3%
		EBRT ± ADT	5 yr BCRFS: 22.8%; 5 yr DMFS: 41.7%; 5 yr PCSS: 99.2%; 5 yr OS: 92.1%;
Markovina (2017) [22]	DMFS, OS	RP ± RT ± ADT	5 yr DM: 33%
		EBRT ± ADT	5 yr DM: 8.9%; EBRT vs RP: DMFS: HR (95%CI): 0.23 (0.07–0.71); OS: HR (95%CI): 1.58 (0.56–4.48)
Reichard (2019) [27]	BCR, LR, DMF,OS	RP ± RT ± ADT	5 yr BCR (95%CI): 40.8% (34.6–47.6); 5 yr LR (95%CI): 13.1% (9.3–18.3); 5 yr DMF (95%CI): 6% (3.6–10.2); 5 yr OS (95%CI): 95.7% (92–97.8) RP vs RT & ADT- LR: HR (95%CI): 2.7 (1.0–7.9); DMF: HR (95%CI): 2.5 (0.8–1.8); OS: HR (95%CI): 1.35 (0.4–4.8)
		RT + ADT	5 yr BCR (95%CI): 13.2% (7.0–23.8); 5 yr LR (95%CI): 7.4% (3.1–16.8); 5 yr DMF (95%CI): 7.3% (3.1–16.7); 5 yr OS (95%CI): 98.5% (89.7–99.8)
Robinson (2018) [17]	PCSM	RP	10 yr PCSM: 8.9%
		EBRT ± ADT	10 yr PCSM: 13.7%; RT vs RP HR (95%CI): 1.03 (0.81–1.31)
Tilki (2019) [31]	PCSM, ACM	RP ± EBRT ± ADT	5 yr PCSM (95%CI)—RP: 21.89% (17.07–27.82); RP + EBRT: 3.93% (1.35–11.19); RP + ADT: 27.04% (20.39–35.32) maxRP: 9.83% (3.82–24.02) AHR (95%CI), MaxRT (ref); RP: 2.80 (1.26–6.22); RP + EBRT: 0.52 (:0.14–1.98); RP + ADT: 3.15 (1.32–7.55); maxRP: 1.33 (0.49–3.64) 5 yr ACM (95%CI)—RP: 26.55% (22.02–34.43); RP + EBRT:12.26% (6.58–22.20); RP + ADT: 36.88%(28.53–44.76); MaxRP: 15.85% (8.27–29.19) AHR (95%CI), MaxRT (ref); RP: 1.65 (0.94–2.91); RP + EBRT: 0.70 (0.31–1.57); RP + ADT: 2.33 (1.23–4.42) MaxRP:0.80 (0.36–1.81)
		EBRT + BT + ADT (maxRT)	5 yr PCSM (95%CI): 2.22% (0.91–5.37); 5 yr ACM (95%CI): 6.79% (4.40–10.40)
Tward (2020) [23]	HRQoL (SHIM, AUA-SS, IPSS, SHIM, RFAS after 2003) DMFS, OS	RP ± RT ± ADT	5 yr DMFS: 83.1%; 5 yr OS: 92.8%;
		EBRT + ADT	5 yr DMFS: 74.6%; 5 yr OS: 79.1%
		EBRT + BT + ADT	5 yr DMFS: 94.8%; 5 yr OS: 87.7% DMFS: EBRT + BT + ADT vs EBRT + ADT: AHR: 0.42, p = 0.13; EBRT + BT + ADT vs RP: AHR: 0.46, *p* = 0.11 OS: no significant difference between surgery/RT regimen
Yamamoto (2016) [24]	BCRFS	RP	5 yr BCRFS: 37.3%
		EBRT + ADT	5 yr BCRFS: 81.3% (*p* < 0.001)

*Abbreviations*: *ACM* All-cause mortality, *ADT* Androgen Deprivation Therapy, *BCRFS* Biochemical Recurrence-Free Survival, *BT* Brachytherapy, *cRFS* Clinical Relapse-Free Survival, *DMFS* Distant Metastases-Free Survival, *EBRT* External Beam Radiotherapy, *LR* Local Recurrence, *OM* Overall Mortality, *OS* Overall Survival, *PCSM* Prostate Cancer-Specific Mortality, *PCSS* Prostate Cancer-Specific Survival, *RP* Radical Prostatectomy, *RT* Radiotherapy

## Discussion

Curative treatment options currently recommended for localized high-risk PCa include RP, possibly as part of multi-modal therapy, and radiation based treatment combined with ADT [10]. There is substantial variation between individual hospitals in the utilization of both treatment options that is not explained by patient- and tumor characteristics or patient preferences [32]. The lack of high-level comparative evidence, absence of consensus regarding the optimal treatment for patients with high-risk PCa, the fact that neither treatment is recommended over the other in current guidelines and different definitions of high-risk PCa (e.g. EAU versus NCCN) contribute to this unwarranted clinical variation [33]. In this review, we have summarized the existing comparative evidence in terms of HRQoL, functional and oncological outcomes.

Several systematic reviews and meta-analysis have already been published on this topic, based on which treatment with RP appears to be more favorable in terms of OS and PCSS [1, 34–37]. However, many studies included in these reviews were published in de late 1990’s or early 2000’s and the eligibility criteria used were less stringent (e.g. no requirements were set regarding the radiation dose). Consequently, results have been included from studies in which the treatment(s) used are now considered suboptimal. For example, technological advances in radiation treatment delivery have enabled dose-escalation, which is currently considered the standard-of-care. Dose-escalation and technological advanced are associated with improved BCRFS, DMFS, PCSS, OS and reduced toxicity [38–42]. Regarding RP, the introduction of the robot-assisted procedure and centralization of care in high-volume hospitals are important developments. Although both developments are associated with improved perioperative outcomes, improvements in functional and oncological outcomes (e.g. DMFS, PCSS and OS) have not been demonstrated [43–45].

In the majority of studies included in the current review, a significant difference in oncological outcomes between treatment with RP and EBRT combined with ADT was not observed. In addition, five year OS and PCSS were generally good. Therefore, differences in functional outcomes and HRQoL are arguably important. Few studies reported these outcomes after treatment for high-risk PCa with RP compared to EBRT and ADT. Genitourinary toxicity and sexual dysfunction were reported more frequently after RP while gastrointestinal toxicity and reduced hormonal function were more common after EBRT combined with ADT. Results from studies comparing different surgical approaches (e.g. robot-assisted versus open RP), more often included functional outcomes. In studies specifically focusing on, or with a substantial proportion of, patients with high-risk PCa, erectile function recovery at 12–24 months after RARP was reported in 23–60% of patients with no erectile dysfunction at baseline. Erectile function recovery was defined as no or mild erectile dysfunction (International Index of Erectile Function-5 score ≥ 17) or erections sufficient for intercourse [46–49]. Urinary continence recovery, in most studies defined as the use of 0–1 safety pad per day, was reported in 60.5–95% [46–48, 50]. In patients with high-risk PCa the additional detrimental effect of adjuvant radiation therapy and/or ADT on functional outcomes should also be considered [51]. In trials comparing different radiation regimens, a cumulative 3- to 5-year incidence of grade ≥ 2 and ≥ 3 GU toxicity of 23–41.3% and 3.5–19% was observed after EBRT, respectively. The reported cumulative 3- to 5-year incidence of grade ≥ 2 and ≥ 3 GI toxicity was 12.2–23.4% and 1.4–3.3%. In addition, Rodda et al. reported a cumulative incidence of any pad use 5 years after treatment of 6.3% and retained or recovered erectile function in 45% of patients with adequate erections before treatment. Either the Radiation Therapy Oncology Group-European Organisation for Research and Treatment of Cancer (RTOG-EORTC) scoring criteria or the Late Effects of Normal Tissue-Somatic, Objective, Management, Analytic (LENT-SOMA) scale were used to score GU- and GI-toxicity and most patients included in these trials received (neo)adjuvant androgen deprivation therapy [52–54]. Due to the limited number of studies directly comparing functional outcomes and HRQoL after RP versus EBRT combined with ADT and the use of different measurement methods across studies reporting these outcomes after either treatment, the magnitude of the effect of RP versus EBRT and ADT on functional outcomes and HRQoL remains largely unknown. Future research efforts, should focus on the effect of different treatment options on these outcome measures that are highly relevant to patients. In this regard, combination therapy of EBRT and brachytherapy should also be considered, as favorable oncological outcomes of this treatment combination have been reported [28, 55]. However, patients treated with EBRT and a brachytherapy boost were included in only one of the studies that evaluated functional outcomes and HRQoL after RP versus radiation based treatment [23].

Strengths of this review include the specific focus on functional outcomes and HRQoL after treatment for high-risk PCa. These outcome measures are currently under-reported in this patient group, which is confirmed by the current review. Furthermore the search strategy and eligibility criteria were chosen to provide a comprehensive summary of the available studies applicable to current clinical practice. Limitations include the fact that the studies included in the current review are, except for one, retrospective in nature (either using data retrospectively collected from medical records or using data from existing databases). In addition, the majority of studies were conducted at a single-institution and in many studies there were potential quality issues in the assessment of outcome measures. Although statistical methods were applied to control for potential confounders in most studies, residual and/or unmeasured confounding remains an issue. For example, patients with a better performance status and fewer comorbidities are more likely to be considered eligible for RP, which is supported by the generally younger age of surgically treated patients. Furthermore, inclusion criteria, definitions of high-risk PCa, applied surgical and radiotherapy techniques and use of adjuvant therapies varied within and across studies. Differences in methodology, outcome measures, and the information that was reported further contributed to the heterogeneity of data, precluding meaningful quantitative synthesis and preventing definitive conclusions regarding the optimal treatment for men with high-risk PCa.

## Conclusions

High-level comparative evidence regarding surgery versus radiation-based treatment for high-risk PCa is lacking. Multiple, primarily retrospective, observational studies comparing RP with dose-escalated EBRT and ADT in this patient population have been published. In the majority of studies, no significant differences in oncological outcomes (e.g. DMFS, PCSS and OS) between treatment with RP and EBRT combined with ADT were observed. Studies reporting functional outcomes and HRQoL are very scarce and the magnitude of the effect of RP versus dose-escalated EBRT with ADT on HRQoL and functional outcomes remains largely unknown. Underlining the necessity for RCTs or well-designed observational studies investigating differences in functional outcomes, HRQoL and to a lesser extent oncological outcomes in the high-risk PCa population.

## Supplementary Information

**Additional file 1.** 

## Data Availability

All data generated or analysed during this study are included in this published article (and its supplementary information files).

## Abbreviations

ACM: All-cause mortality
AUA-SS: American Urological Association Symptom Score
ADT: Androgen deprivation therapy
BCRFS: Biochemical recurrence-free survival
BED: Biologically effective dose
BT: Brachytherapy
cRFS: Clinical recurrence-free survival
DMFS: Distant metastasis-free survival
EHR: Electronic health records
EAU: European Association of Urology
EQD2: Equivalent dose 2 Gy fractions
EPIC: Expanded Prostate Cancer Index Composite
EBRT: External beam radiotherapy
GI: Gastrointestinal
GU: Genitourinary
HRQoL: Health-related quality of life
IPSS: International Prognostic Scoring System
ISUP: International Society of Urological Pathology
ISRCTN: International Standard Randomized Controlled Trial Number
LRP: Laparoscopic radical prostatectomy
LENT-SOMA: Late Effects of Normal Tissue-Somatic, Objective, Management, Analytic
LR: Local recurrence
MCID: Minimal clinically important difference
NCCN: National Comprehensive Cancer Network
OM: Overall mortality
OS: Overall survival
PROMS: Patient reported outcome measures
PLND: Pelvic lymph node dissection
PRIMSA: Preferred Reporting Items for Systematic Reviews and Meta-analyses
PCa: Prostate cancer
PCSM: PCa-specific mortality
PCSS: PCa-specific survival
ProtecT: Prostate Testing for Cancer and Treatment
RP: Radical prostatectomy
RTOG-EORTC: Radiation Therapy Oncology Group-European Organisation for Research and Treatment of Cancer
RT: Radiotherapy
RCT: Randomized controlled trial
RFAS: Rectal Function Assessment Scale
RARP: Robot-assisted radical prostatectomy
SPCG-15: Scandinavian Surgery Versus Radiotherapy for Locally Advanced Prostate Cancer
SHIM: Sexual Health Inventory in Men
SF-36: Short Form-36
3D-CRT: Three-dimensional conformal radiotherapy
VMAT: Volumetric arc therapy
